# Forsythiaside A ameliorates sepsis-induced acute kidney injury via anti-inflammation and antiapoptotic effects by regulating endoplasmic reticulum stress

**DOI:** 10.1186/s12906-023-03855-7

**Published:** 2023-02-03

**Authors:** Yi Chen, Wei Wei, Jingnan Fu, Teng Zhang, Jie Zhao, Tao Ma

**Affiliations:** 1 Department of General Surgery, Tianjin Medical University General Hospital, 154 Anshan Road, Heping District, Tianjin, 300052 China; 2 State Key Laboratory of Integrated Traditional Chinese and Western Medicine, General Hospital of Tianjin Medical University, Tianjin, 300052 China; 3 Department of Emergency Medicine, Tianjin Medical University General Hospital, Tianjin, 300052 People’s Republic of China; 4 Department of Respiratory and Intensive Care Medicine, Tianjin Medical University General Hospital, 154 Anshan Road, Heping District, Tianjin, 300052 China

**Keywords:** Sepsis, Acute kidney injury, Forsythiaside A, ER stress, Inflammation

## Abstract

**Ethnopharmacological relevance:**

Sepsis is a systemic inflammatory response syndrome caused by an infection in the body, and accompanying acute kidney injury (AKI) is a common complication of sepsis. It is associated with increased mortality and morbidity. Forsythia Fructus, the dried fruit of Forsythia suspensa (Thunb.) Vahl, is a commonly used traditional Chinese medicine.

**Aims of the study:**

This study aimed to elucidate the protective effect of Forsythiaside A (FTA) on sepsis-induced AKI by downregulating inflammatory and apoptotic responses, and exploring its underlying mechanism.

**Methods:**

Septic AKI was induced through intraperitoneal injection of LPS (10 mg/kg) using male C57BL/6 mice and pretreated with FTA or control saline. First, we assessed the degree of renal injury by creatinine, blood urea nitrogen measurement, and HE staining of renal tissue; secondly, the inflammation and apoptosis were measured byELISA, qPCR, and TUNEL immunofluorescence; finally, the mechanism was explored by computer molecular docking and Western blot.

**Results:**

Our data showed that FTA markedly attenuated pathological kidney injuries, alleviated the elevation of serum BUN and Creatinine, suggesting the renal protective effect of FTA. Notably, FTA significantly inhibited the renal expression of proinflammatory cytokine IL-1β, IL-6, and TNF-α both at protein and mRNA levels and attenuated cell apoptosis in the kidney, as measured by caspase-3 immunoblot and TUNEL assay, indicating its anti-Inflammation and antiapoptotic properties. Mechanistically, administration of LPS resulted in robust endoplasmic reticulum (ER) stress responses in the kidney, evidenced by glucose-regulated protein 78(GRP78) upregulation, protein kinase RNA–like endoplasmic reticulum kinase (PERK) activation, eukaryotic initiation factor 2 alpha (elF2α) phosphorylation and C/EBP homologous protein (CHOP) overexpression, which could be significantly blocked by FTA pretreatment. Dynamic simulation and molecular docking were performed to provide further insight.

**Conclusions:**

Collectively, our data suggest that FTA ameliorates sepsis-induced acute kidney injury via its anti-inflammation and antiapoptotic properties by regulating PERK signaling dependent ER stress responses.

**Supplementary Information:**

The online version contains supplementary material available at 10.1186/s12906-023-03855-7.

## Introduction

Sepsis is a systemic inflammatory response syndrome characterized by organ dysfunction caused by a dysregulated patient response to infection [1]. Acute kidney injury (AKI) is a common and potentially fatal complication of sepsis, occurring in approximately 50% of patients with severe sepsis [2, 3]. It is generally believed that the direct trigger of septic AKI is the overwhelming inflammatory response caused by sepsis, which causes the body to produce and release large amounts of cytokines, such as interleukin-1β (IL-1β), interleukin-6 (IL-6), tumor necrosis factor-α (TNF-α), chemokines, and inflammatory mediators, which ultimately lead to renal tubular epithelial cell damage [4].

Numerous studies have demonstrated that extensive apoptosis occur in the pathophysiology process of sepsis-induced AKI [5, 6]. A protective stress response of cells, called endoplasmic reticulum (ER) stress, is important and widespread during sepsis-induced AKI [7, 8]. ER stress is closely related to the process of apoptosis. Under endoplasmic reticulum (ER) homeostasis, GRP78 protein (glucose-regulated protein–78, also known as binding immunoglobulin protein or BiP) binds to the ER stress sensor and maintains it in an inactive state [9]. During sepsis, the ER accumulates misfolded and unfolded proteins that have a higher affinity for GRP78 proteins, so ER stress sensors are released, activating ER stress and triggering the unfolded protein response (UPR) [10, 11]. Double-stranded RNA-dependent protein kinase (PKR)-like ER kinase (PERK) is one of the stress sensors and a major initiator of ER stress. PERK dissociates from GRP78 to trigger the UPR and a series of downstream proteins in the signaling pathway, including phosphorylation α subunit of eukaryotic initiation factor-2 (eIF-2α), activating transcription factor 4 (ATF4), and C/EBP homologous protein (CHOP) [12]. CHOP is a well-known mediator of ER stress-mediated cell death due to its ability to activate numerous pro-apoptotic factors [13, 14]. Therefore, therapeutic strategies aimed at inhibiting ER stress-induced apoptosis bear the potential to treat sepsis-induced AKI.

Forsythia is a traditional Chinese medicine, recorded in the Shennong Bencao Jing for the treatment of fever, inflammation, gonorrhea, carbuncle, and erysipelas [15, 16]. Modern pharmacology shows that Forsythiaside A (FTA) extracted from Forsythia Fructus has antibacterial [17], antioxidant [18], antiviral [19, 20], hepatoprotective [21, 22], anti-inflammatory [23, 24], neuroprotective [25] and other pharmacological effects. Numerous experimental studies have reported the therapeutic effects of forsythoside A in various models of inflammation. For example, FTA was found to exert anti-inflammatory effects by inhibiting the production of IL-6, IL-1β, TNF-α and COX-2 through the NF-κB pathway in LPS-induced injury on the bursa of Fabricius of chickens [26]. FTA alleviates zymosan-induced acute peritonitis [27]. FTA improves Staphylococcus aureus-induced inflammation in primary bovine mammary epithelial cells by inhibiting MARK signaling pathway [28]. FTA protects mice from cigarette smoke-induced lung injury by inhibiting NF-κB and activating the Nrf2 signaling pathway [29]. However, in sepsis-induced AKI, there have been few studies on whether FTA can protect the kidneys and improve kidney damage.

Therefore, we hypothesized that forsythiaside A could ameliorate sepsis-induced AKI and attempted to demonstrate this by establishing an animal model of septic AKI induced by intraperitoneal injection of LPS. Additionally, we examined whether ER stress plays an important role in the underlying mechanism of this protective effect and provided some recommendations for the use of forsythiaside A in clinical practice.

## Materials and Methods

### Animals

Wild-type C57BL/6 male mice (6–8 weeks) were purchased from Vital River Laboratories (Beijing, China) and caged at a constant temperature of 20–24 °C and humidity of 60–70% with a 12-h light/dark cycle. We randomly divided mice into three experimental groups: sham, LPS, and FTA + LPS groups. The LPS and FTA + LPS groups were i.p. injected with LPS (10 mg/kg). One hour before LPS injection, mice in the FTA + LPS group were intraperitoneally injected with forsythiaside A (40 mg/kg). Mice in the sham or LPS groups were given the same volume of saline (i.p.). After twenty four hours, the mice were anesthetized with isoflurane and sacrificed, blood was collected from the heart, and kidney tissue was perfused and collected. All animal experiments in this study were approved and supervised by the Ethics Committee of Tianjin Medical University General Hospital (approval no. IRB2022-DWFL-44).

### Renal function analysis

Animal blood samples were collected by direct cardiac puncture under deep anesthesia and centrifuged at 1500 × g for 15 min at 4 °C, followed by supernatant collection. We use a mindray BS240VET automatic biochemical analyzer to detect the levels of BUN and CRE in renal function.

### Histopathological examination

Twenty-four hours after all intraperitoneal injections were performed, we gathered kidney tissue samples and immediately fixed them in a 10% formalin solution. Then, kidney tissues were dehydrated in graded ethanol, embedded in paraffin, cut into 5 μm thick slices, and stained with hematoxylin and eosin reagent (H&E). Images were obtained with an optical microscope (3DHISTECH Pannoramic MIDI). Without knowledge of the assigned group, the pathologist evaluating the images randomly selected five different regions to observe in each sample. The degree of renal histopathological damage after sepsis was assessed according to renal interstitial congestion and edema, intraluminal cell swelling, cell necrosis, and protein casts. Renal injury was assessed using the following 5-point scoring system: 0—normal kidney morphology, 1—renal tissue damage ≤ 10%, 2—renal tissue damage 11–25%, 3—renal tissue damage 26–45%, 4 points—renal tissue damage 46–75%, 5 points—renal tissue damage ≥ 76%.

### Inflammatory factor detection

Kidney tissues collected from different groups were homogenized with equal volumes of PBS on ice, and supernatants were collected after centrifugation at 12,000 rpm for 15 min at 4 °C. We performed ELISA assays to detect levels of inflammatory cytokines in kidney tissues according to the manufacturer’s protocols. Tumor necrosis factor (TNF-α), interleukin 1β (IL-1β), and interleukin 6 (IL-6) enzyme-linked immunosorbent assay (ELISA) kits were purchased from DAKEWE Biotech Co., Ltd.

First, we also detected the levels of these inflammatory factors in kidney tissue using a quantitative real-time polymerase chain reaction (qPCR). We dissolved kidney tissues in TRIzol reagent (TF201-50, TIANMOBIO) to extract the total RNA. Next, we quantified and confirmed the concentration and purity of total RNA by spectrophotometry at 260 nm and 280 nm, respectively. RNA was then converted to cDNA using the ProtoScript First Strand cDNA Synthesis Kit (NEB). We measured mRNA expression using the instrument CFX96 Deep Well Dx ORM (BIO-RAD) and used the primer sets described in Table 1. Each cDNA sample was analyzed in triplicate. We analyzed the relative mRNA expression using the 2–∆∆Ct method, normalized using GAPDH expression.

**Table 1:** Genes’ primer sequence

Genes	Forward primers	Reverse primers
IL- 1β	5′-TCGCAGCAGCACATCAACAAGAG-3′	5′-AGGTCCACGGGAAAGACACAGG-3′
IL-6	5′-TAGTCCTTCCTACCCCAATTTCC-3′	5′-TTGGTCCTTAGCCACTCCTTC-3′
TNF- α	5′-GACGTGGAACTGGCAGAAGAG-3′	5′-TTGGTGGTTTGTGAGTGTGAG-3′
GAPDH	5′-ACTCCACTCACGGCAAATTC-3′	5′-TCTCCATGGTGGTGAAGACA-3′

### Western blot assay

We assessed the regulatory effects of forsythiaside A on apoptosis and ER stress by examining protein expression levels by western blotting. We took the same weight of kidney tissue from each group, added tissue lysis buffer and protease inhibitors, and homogenized them at 4 ℃ for total protein extraction. Protein concentrations were determined using a BCA protein assay kit. An equivalent of 50 μg of protein was loaded onto a gel for sodium dodecyl sulfate–polyacrylamide gel electrophoresis (SDS-PAGE) and then transferred to a polyvinylidene difluoride (PVDF) membrane. After blocking with 5% nonfat milk, the resulting blot was incubated with different primary antibodies against the relevant proteins at 4 °C overnight. GRP78 antibody (ab108613) was purchased from Abcam (Cambridge, UK). Caspase-3 (#9661), PERK (#3192), eIF 2α (#9722), p-eIF 2 α (#3398) and CHOP (#2895), purchased from CST Company (Cell Signaling Technology, USA), were used during our experiments. The ATF4 antibody (60,035) was purchased from Proteintech Group, Inc. Anti-β-actin antibodies (c1313, APPLYGEN, China) were used to quantify the protein expression. After incubation with anti-rabbit and anti-mouse HRP-conjugated secondary antibodies (#7074 s and #7076 s, respectively, Cell Signaling Technology, USA) at room temperature for 1 h, PVDF membranes were washed three times. The blots were visualized using BLT GelView 6000 Pro Chemiluminescence Imaging System. The relative band intensity was quantified by ImageJ software.

### Exploring the molecular mechanism for the inhibitory effect of forsythiaside A on ER stress

Elucidating the geometric and energy matching relationships between the molecular structure of drugs and specific proteins in organisms can help us understand the molecular mechanisms of drug effectiveness. Therefore, we used an in silico docking method to explore the molecular mechanism for the inhibitory effect of forsythiaside A on ER stress. In the MODELLER9v14 program, we constructed a homology model of GRP78 by a homology modeling method. We utilized Autodock Version 4.2 to dock the flexible structure of forsythiaside A into the cavity of GRP78. The protein domain was kept rigid. We added polar hydrogen atoms to GRP78, incorporated non-polar hydrogen atoms, and built a grid box covering the entire protein for blind docking. The molecular docking study for the binding of forsythiaside A to GRP78 was carried out by a Lamarckian Genetic Algorithm (LGA) method. The number of runs was set to 50. In geometric matching, we analyzed the interaction between forsythoside and GRP78, including hydrophobic contacts and hydrogen bonding. In energy matching, we analyzed the optimal conformation with the lowest docking energy between forsythiaside and GRP78.

### Statistical analysis

All data were analyzed using SPSS 21.0 statistical software or GraphPad Prism 8.0. All data are expressed as mean ± standard deviation, and the differences between groups were compared by one-way OneWay ANOVA analysis (multiple groups), and *P* < 0.05 was considered statistically significant.

## Result

### Forsythiaside A ameliorated LPS-induced renal dysfunction and histopathological abnormalities

**Fig. 1:**
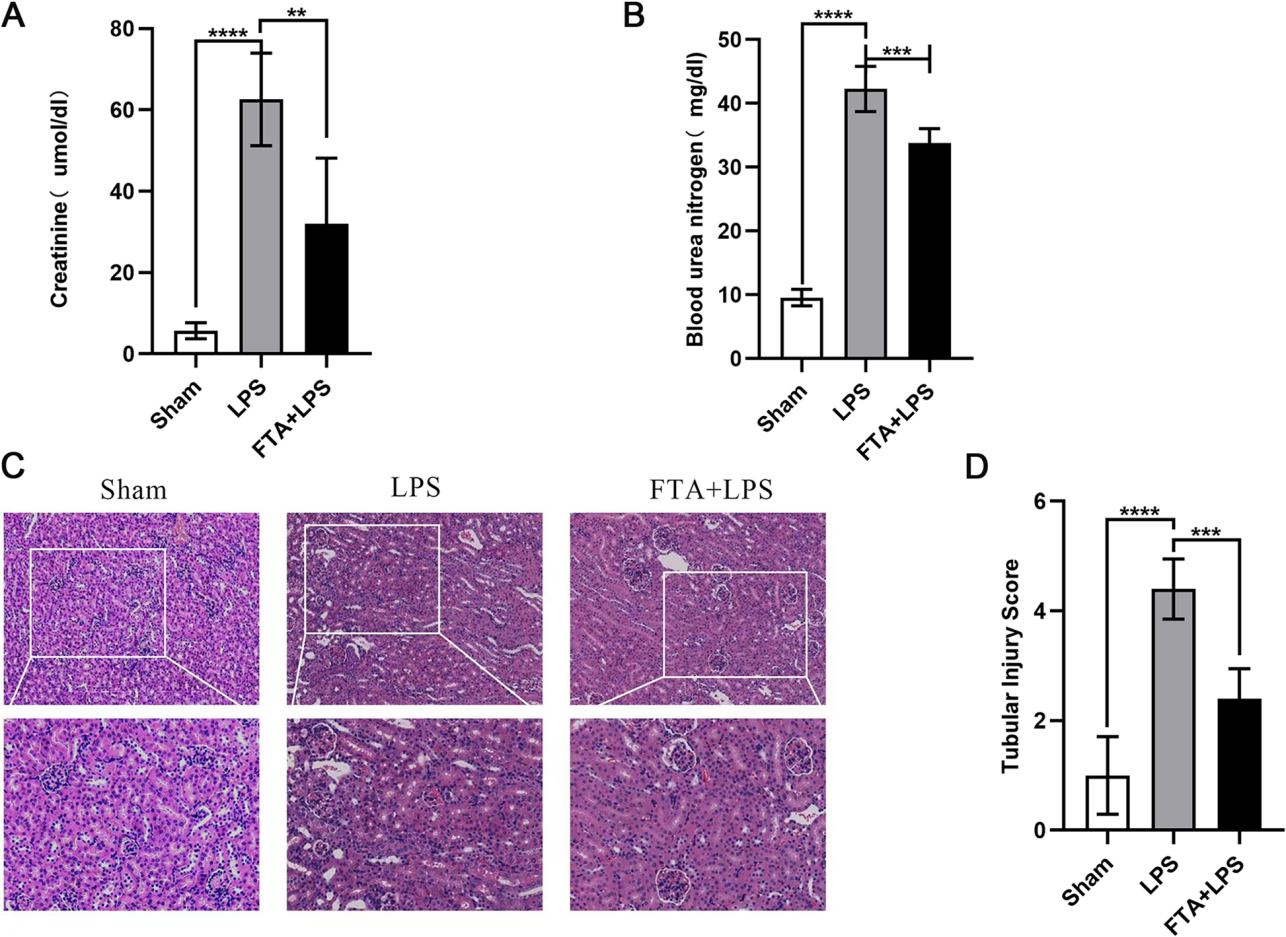
Forsythiaside A protected kidney function in LPS-Induced AKI mice. **A** The blood urea nitrogen level in mice serum. **B** The creatinine level in mice serum. Forsythiaside A attenuated histopathological damage in the kidneys of LPS-Induced AKI mice. **C** 24 h after intraperitoneal injection of LPS, H&E staining was performed on kidney tissue sections to observe kidney morphology. **D** Scoring of renal tubular damage according to a 5-point scoring system. Representative data of five individual samples in each group. original magnification, 40 × and 100x. ∗ *P* < 0.05, ∗  ∗ *P* < 0.01, and ∗  ∗  ∗ *P* < 0.001

First, we investigated whether forsythiaside A could improve renal insufficiency during sepsis. Blood urea nitrogen (BUN) and serum creatinine (CRE) are two widely used indicators for assessing renal function. After LPS (i.p.) in C57BL/6 mice, we observed increased levels of BUN and CRE, which confirmed the successful establishment of the sepsis-induced AKI model (Fig. 1A and B). t CRE (*P* < 0.01) and BUN (*P* < 0.001) levels in the FTA + LPS group were significantly lower than in the LPS group. We then looked at histopathological lesions between the different groups. We performed H&E staining on kidney tissue sections. In the sham group, we observed few renal histopathological changes. In the renal tissue sections of the LPS group, we observed obvious cell swelling, degeneration and necrosis of some cells, a disorder of glomerular blood vessels, and scattered inflammatory cell infiltration (*P* < 0.0001). However, the FTA + LPS group only showed minor damage, including small cell swelling and brush border damage (Fig. 1C). The tubular injury score also showed that FTA significantly attenuated LPS-induced histopathological changes (*P* < 0.001) (Fig. 1D).

### Anti-inflammatory effect of forsythiaside A on sepsis-induced AKI

**Fig. 2:**
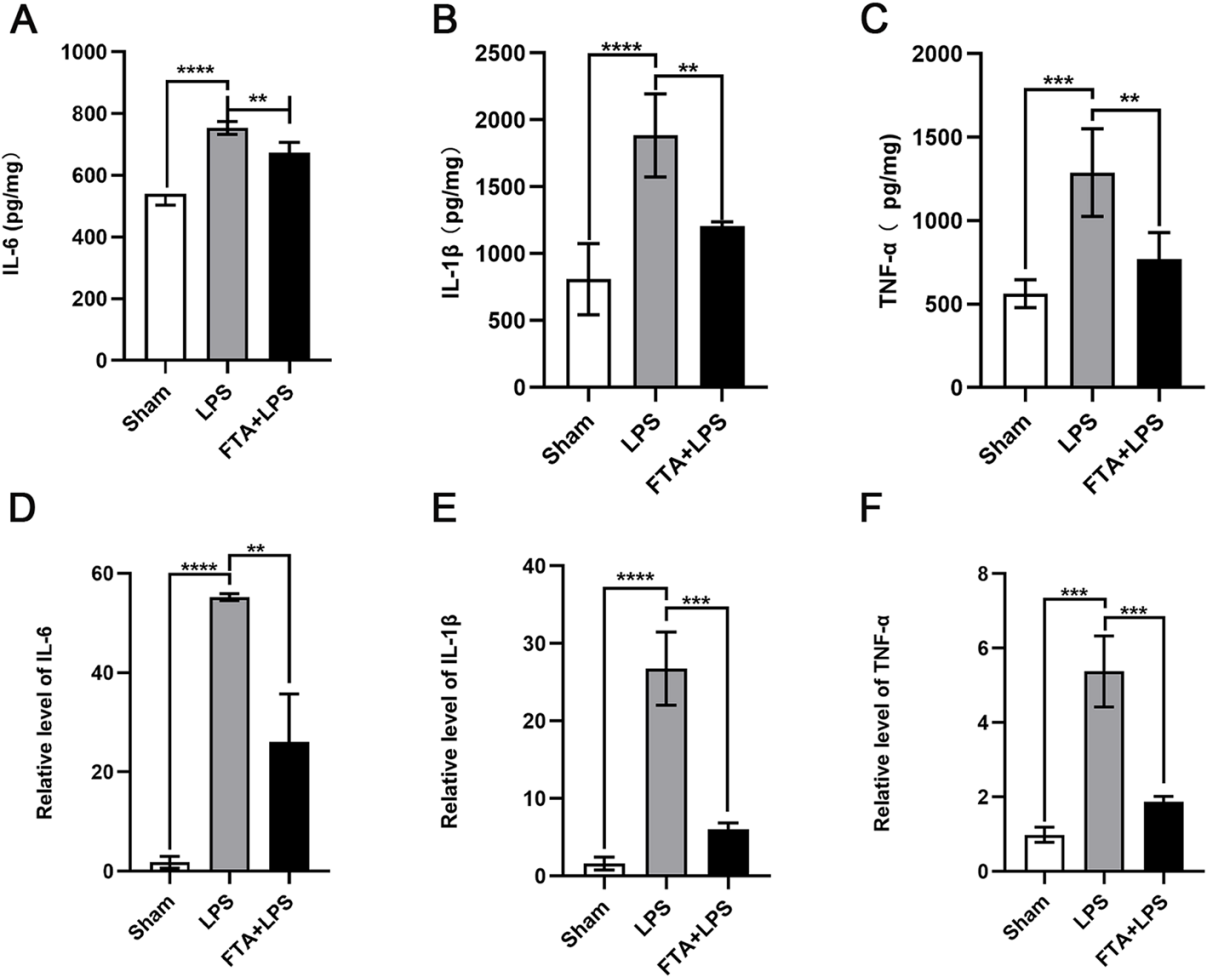
Forsythiaside A inhibited the inflammatory response in the kidney after intraperitoneal injection of LPS. The levels of **A** IL-6, **B** IL-1β, and **C** TNF-α in kidney were assessed by ELISA. The levels of **D** IL-6, **E** IL-1β, and **F** TNF-α mRNA in kidney were assessed by qPCR. ∗ *P* < 0.05, ∗  ∗ *P* < 0.01, and ∗  ∗  ∗ *P* < 0.001

In sepsis-induced kidney injury, many pro-inflammatory cytokines are released, mainly IL-1β, IL-6, and TNF-α. We sought to investigate the effect of forsythiaside A on these pro-inflammatory cytokines and measured their levels in the kidneys of different groups by ELISA and their mRNA expression by qPCR. The injection of LPS significantly increased the expression of IL-1β (*P* < 0.0001), IL-6 (*P* < 0.0001), and TNF-α (*P* < 0.001) in the kidney (Fig. 2A-C). The levels of pro-inflammatory cytokines in the FTA + LPS group were decreased compared with those in the LPS group, confirming the anti-inflammatory effect of FTA (*P* < 0.01). The results of Real-time qPCR are the same as above (Fig. 2D-F). The expressions of inflammatory factors in the LPS group were significantly increased, indicating that a significant inflammatory reaction occurred in the LPS group (IL-1β (*P* < 0.0001), IL-6 (*P* < 0.0001), and TNF-α (*P* < 0.001)). In contrast, the expression of inflammatory factors in the FTA group was decreased, suggesting that FTA has an inhibitory effect on inflammation (IL-1β (*P* < 0.001), IL-6 (*P* < 0.01), and TNF-α (*P* < 0.001)).

### Antiapoptosis effects of forsythiaside A on Sepsis-induced AKI

**Fig. 3:**
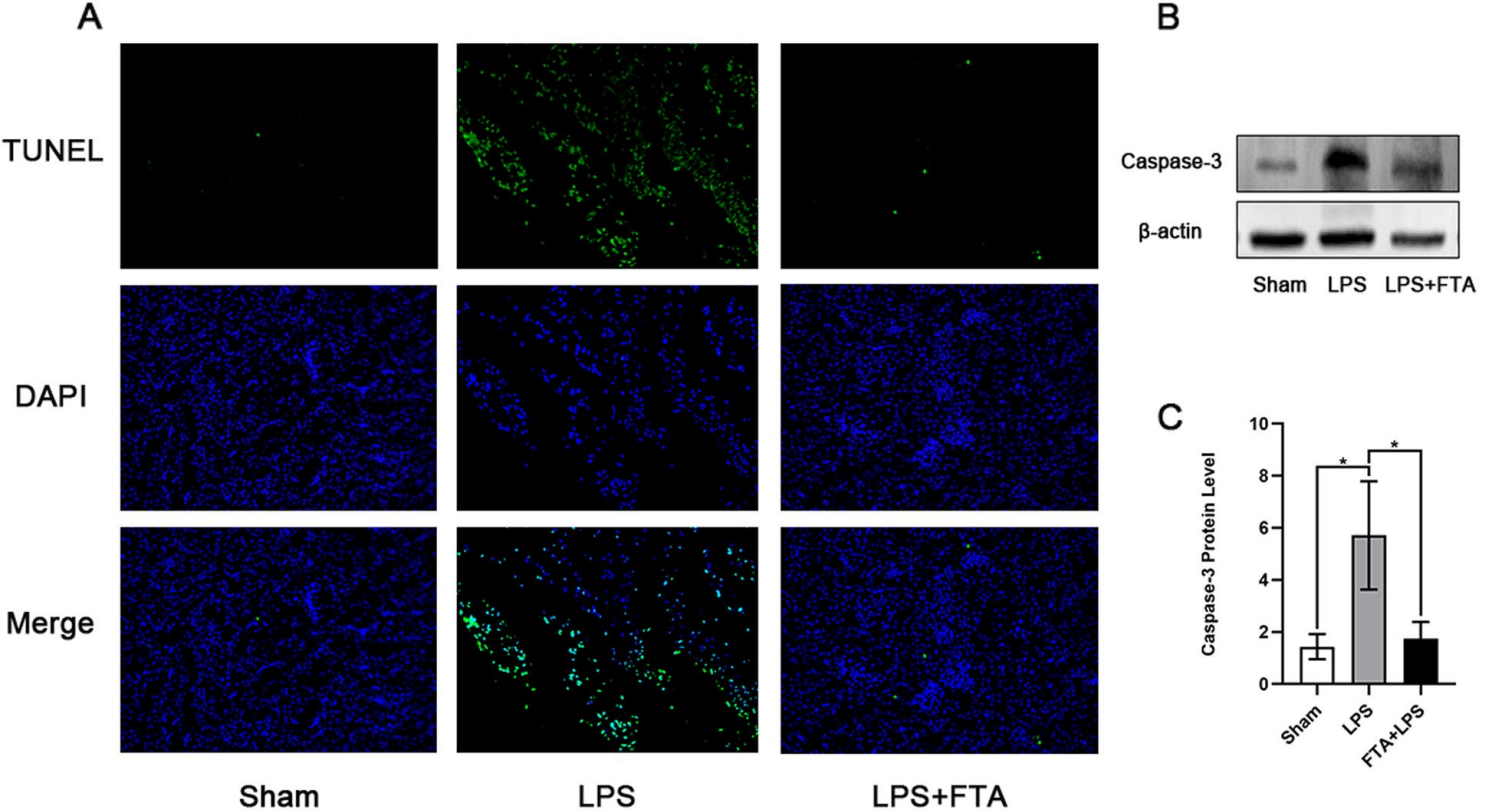
Antiapoptosis effects of forsythiaside A on Sepsis-Induced AKI. **A** TUNEL green fluorescence assay was performed on kidney tissue sections. **B** The kidney protein expression levels of Caspase-3 was detected by Western blot, and the relative band intensities (fold of the sham group) were shown in (**C**). Representative sections; original magnification, 100x. ∗ *P* < 0.05, ∗  ∗ *P* < 0.01, and ∗  ∗  ∗ *P* < 0.001

The apoptosis of renal tubular cells in renal tissue is an important part of the development of septic AKI, as it can lead to significant damage to the septic kidney. Therefore, we performed the TUNEL (TdT-mediated dUTP Nick-End Labeling) assay to evaluate the apoptosis of renal cells in each group of kidney tissues. As shown in Fig. 3A, we observed a few green fluorescently labeled TUNEL-positive cells in the kidney tissue of the sham group, while the number of positive cells was significantly increased after LPS injection. Notably, the proportion of TUNEL-positive cells was significantly decreased in the FTA + LPS group compared with that in the LPS group. This indicated that FTA treatment significantly reduced apoptosis in renal tissue. We then examined the levels of cleaved caspase-3 in the kidneys, and the results were consistent with the TUNEL assay results. The LPS (i.p.) also resulted in a significant increase in caspase-3 (*P* < 0.05), and forsythiaside A treatment reduces caspase-3 protein expression (*P* < 0.05) (Fig. 3B and C). In conclusion, renal cells undergo significant apoptosis activity during sepsis, which can be reduced by forsythiaside A treatment.

### Forsythiaside A attenuated the ER stress-related apoptosis process on sepsis-induced AKI

**Fig. 4:**
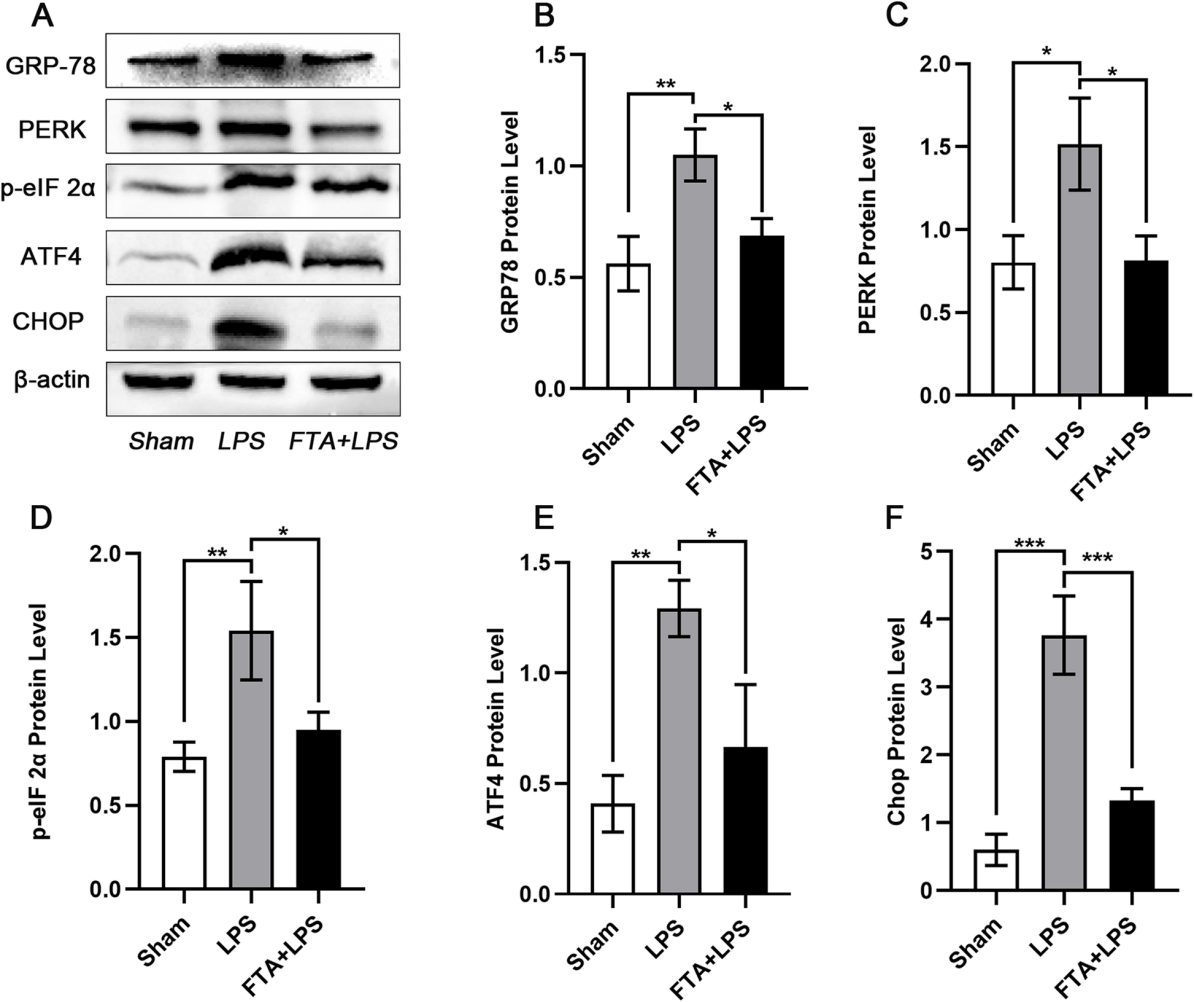
Forsythiaside A Attenuated the ER Stress-related Apoptosis Process on Sepsis-induced AKI. **A** Western blot bands of GRP78, PERK, p-eIF 2α, ATF4 and CHOP in kidney tissues. Western blot analysis and quantification of **B** GRP78, **C** PERK, **D** p-eIF 2α, **E** ATF4 and **F** CHOP

ER stress is an extremely important pathophysiological process in sepsis-induced AKI. Therefore, we examined important markers of ER stress and proteins on the ER stress signaling pathway associated with apoptosis. We examined the levels of GRP78/PERK/p-eIF 2α/ATF4/CHOP, which are involved in regulating the UPR response and are closely related to apoptosis and tissue damage in the kidney, by western blot (Fig. 4). Compared to those in the sham group, the levels of these proteins all tended to increase in the LPS group (GRP78 (*P* < 0.01) PERK (*P* < 0.05) p-eIF 2α (*P* < 0.01) ATF4 (*P* < 0.01) CHOP (*P* < 0.001)), implying the activation of ER stress. There was a significant downward trend in the FTA + LPS group(GRP78 (*P* < 0.05) PERK (*P* < 0.05) p-eIF 2α (*P* < 0.05) ATF4 (*P* < 0.05) CHOP (*P* < 0.001)), indicating that forsythiaside A may down-regulate the ER stress-related apoptotic process.

### The molecular mechanism for the inhibitory effect of forsythiaside A towards ER stress

**Fig. 5:**
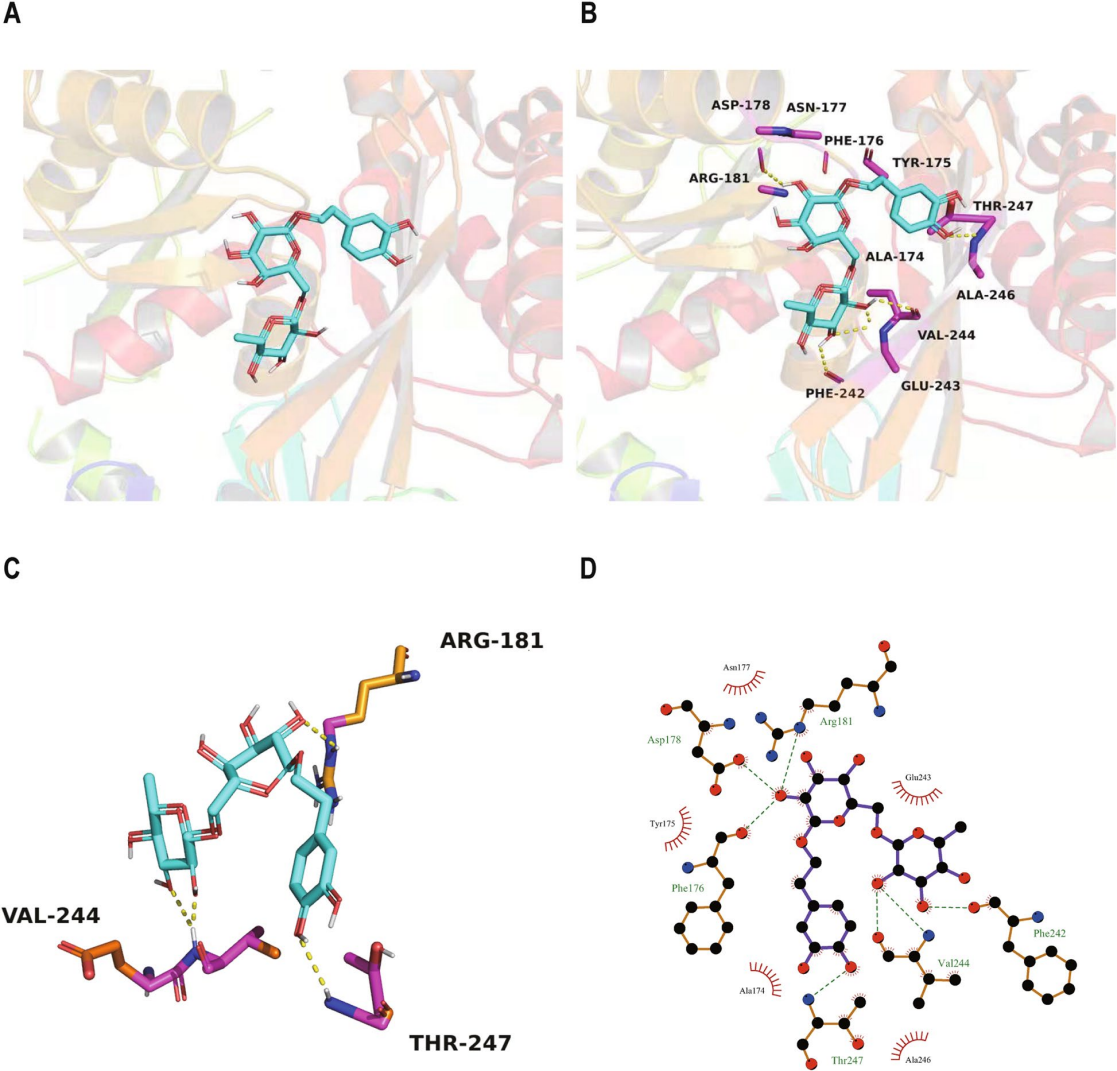
**A** Activity pocket of GRP78 binding with Forsythiaside A. **B** The active site of GRP78 binding with Forsythiaside A was composed of amino acids residues Asp-178, Asn-177, Phe-176, Tyr-175, Arg-181, Thr-247, Ala-174, Ala-246, Val-244, Phe-242, Glu-243. **C** The interaction between Forsythiaside A with the activity cavity of GRP78. **D** In the binding pocket of GRP78, three hydrogen bonds with amino acids Arg-181、Val-244、Thr-247. Forsythiaside A formed hydrophobic contacts with two amino acids residues (Ala-174, Ala-246) in the active pocket of GRP78

We found the reason for the inhibitory effect of forsythiaside A on sepsis-induced ER stress from the perspective of bioinformatics. We determined the interaction mode of forsythiaside A with the SBD of GRP78 using molecular docking analysis (Fig. 5A and B). Interestingly, we found that there is indeed some interaction between the active site residues of GRP78 and forsythoside, with a binding energy of -1.97 kcal/mol in its top binding pose. The lower the score, the stronger the binding affinity. The ability of forsythiaside A to inhibit GRP78 depends on its structurally related binding affinity to GRP78. This shows that forsythiaside A has a good binding ability to GRP78.

Hydrogen bonding and hydrophobic interactions stabilize the interaction between GRP78 and forsythiaside A. During the molecular docking simulation of GRP78 and forsythiaside A, we found that three residues (Arg-181, Val-244, Thr-247) in the active site of GRP78 are involved in the hydrogen bond formation with forsythiaside A (Fig. 5C). In addition, hydrophobic interactions (Ala-174, Ala-246) play an important role in the binding of GRP78 to forsythiaside A (Fig. 5D). The results showed that forsythiaside A formed favorable interactions with the active conformation of GRP78.

## Discussion

Sepsis-related AKI is considered one of the most severe complications after sepsis due to its high mortality rate. However, treatment options for sepsis-related AKI are still limited and mortality remains high, making it a challenging issue for healthcare workers in their clinical practice. According to some previous studies, inflammation is considered a key trigger for sepsis-related AKI, and apoptosis is an important component of sepsis-related AKI [30]. Numerous experimental studies have focused on the therapeutic effects of forsythoside A on various inflammatory diseases, such as acute peritonitis [27] and pulmonary inflammation [29]. In addition, studies have shown that forsythiaside A has a protective effect on apoptosis [25, 31]. Therefore, it is of great clinical significance to elucidate the renal protective effect of forsythiaside A on sepsis and explore its underlying mechanism.

We created a sepsis-induced AKI mouse model by the intraperitoneal injection of LPS and strictly set control and drug pre-treatment groups, collecting blood and kidney tissue samples 24 h later. Measurement of serum urea and creatinine levels is widely used to determine the degree of kidney toxicity [32]. First, we detected the levels of BUN and CRE in the blood of mice to evaluate the renal function of each group. These values were significantly increased after LPS injection, indicating that the sepsis-induced AKI model was successfully established. The levels of BUN and CRE in mice treated with FTA were significantly decreased. FTA exhibited kidney protective effect in a previous study. Chan Lu et al. found that in a rat model of adriamycin-induced nephropathy, FTA treatment significantly reduced CRE and BUN levels and alleviated renal dysfunction in adriamycin-induced nephropathy rats [33]. This finding is consistent with our study indication protective role of FTA in LPS-induced kidney injury, and we subsequently confirmed the results using renal histopathological damage analysis. We next sought to explore which parts of the set of pathophysiological processes in sepsis-induced AKI are affected by FTA.

Three pro-inflammatory cytokines, IL-1β, IL-6 and TNF-α, have attracted much attention due to their important roles in inflammation-related diseases. In a previous study of LPS-induced acute mastitis in mice, a significant reduction in the expression of these factors and a significant reduction in inflammatory symptoms was observed after the FTA intervention [34]. In addition, Chunyan Liu et al. found that the levels of these factors in the lung tissue of mice with acute lung injury were significantly reduced after the administration of FTA in combination with other compounds [35]. In our study, we examined the levels of major pro-inflammatory cytokines between different groups and found that IL-1β, IL-6, and TNF-α were significantly decreased at both RNA and protein levels in the FTA + LPS group compared with the LPS group. In parallel to data obtained in other studies, the results of our study indicated that FTA treatment is beneficial to suppress the inflammatory response in the kidneys. It has been reported that the ratio of Bcl-2/Bax in skin cells of mice treated by FTA increased by 60%, and FTA reduced the expression of caspase-3 to inhibit apoptosis [36]. Caspase-3 is the most critical apoptosis executor downstream of Bcl-2 and Bax. We then observed attenuated apoptosis in the kidney by caspase-3 immunoblotting and the TUNEL assay, suggesting the anti-apoptotic properties of FTA treatment. Thus, we revealed that FTA treatment ameliorated sepsis-induced AKI mediated by its anti-inflammatory and anti-apoptotic effects.

Endoplasmic reticulum (ER) stress is a protective stress response of cells, mainly triggering the UPR response in response to misfolded and unfolded protein aggregation in the ER lumen. Transient and mild ER stress-induced UPR signaling is an adaptive maintenance mechanism of the body. However, prolonged ER stress no longer shows a protective effect and induces endogenous apoptosis, which ultimately affects the outcome of stressed cells, such as adaptation, injury, or apoptosis [37, 38]. ER stress-mediated apoptosis eliminates irreversibly disorganized cells, which is an important factor in the pathogenesis of renal disease [39]. In mouse and rat models of renal ischemia–reperfusion injury, a pharmacological inhibitor of ER stress (i.e., Taurosodeoxycholic acid-TUDCA) inhibits renal tissue damage and tubular cell death, thereby providing protection against AKI [40]. Therefore, drugs targeting ER stress may provide treatment opportunities for AKI. In a previous study on focal cerebral ischemia, FTA treatment reduced the expression of ER stress-related proteins in rat brain tissue [41]. Therefore, we focused on the level of apoptosis associated with ER stress in the kidney. We found that intraperitoneal injection of LPS resulted in a strong endoplasmic reticulum stress response in the kidney, as evidenced by GRP78 upregulation, PERK activation, elF2α phosphorylation, and CHOP overexpression, which could be significantly blocked by FTA pretreatment. GRP78 is a major regulator of UPR in response to ER stress and has an affinity for attaching to unfolded proteins. It has been reported that a cyclic peptide can target the GRP78 binding site to prevent cancer virulence. Therefore, we have a bold guess whether FTA plays a role through the combination with GRP78 [42, 43]. So we modeled the interaction between forsythoside A and GRP78 by molecular docking to predict the binding mode and affinity. The results showed that forsythiaside A formed favorable interactions with the active conformation of GRP78. The negative binding energy indicates that spontaneous bonding can occur between them, and hydrogen bonding and hydrophobic action stabilize the bonding between them. This may be related to this traditional Chinese medicine down-regulating the expression of GRP78 and inhibiting the ER stress signaling pathway. We speculate that it is entirely possible that forsythiaside A could target GRP78 and then either reduce its production or stabilize it to reduce binding to misfolded and unfolded proteins. The possible mechanism of FTA's action on GRP78 suggested by molecular docking needs to be verified in vivo and in vitro experiments.

In conclusion, in a mouse model of LPS-induced renal injury, we found that FTA could establish renal protection in the early stage of sepsis and improve renal inflammation and apoptosis. FTA also acts as a potential inhibitor of ER stress-related apoptosis, and its regulatory effect is mediated in part through inhibition of the PERK pathway. Based on our findings, I speculate that FTA may be a promising candidate for the treatment of sepsis-induced AKI.

## Supplementary Information

**Additional file 1.**

## Data Availability

The datasets used and/or analyzed during the current study are available from the corresponding author upon reasonable request.

## Abbreviations

AKI: Acute kidney injury
FTA: Forsythiaside A
ER: Endoplasmic reticulum
GRP78: Glucose-regulated protein 78
PERK: Protein kinase RNA–like endoplasmic reticulum kinase
elF2α: Eukaryotic initiation factor 2 alpha
CHOP: C/EBP homologous protein
ATF4: Activating transcription factor 4
IL-1β: Interleukin-1β
IL-6: Interleukin-6
TNF-α: Tumor necrosis factor-α
UPR: Unfolded protein response
LGA: Lamarckian Genetic Algorithm
BUN: Blood urea nitrogen
CRE: Serum creatinine
