# Supplementary material for: Forsythiaside A ameliorates sepsis-induced acute kidney injury via anti-inflammation and antiapoptotic effects by regulating endoplasmic reticulum stress
Source: BMC Complement Med Ther. 2023 Feb 3;23:35. doi: 10.1186/s12906-023-03855-7 (PMC9896724; doi:10.1186/s12906-023-03855-7)

Figure 3B

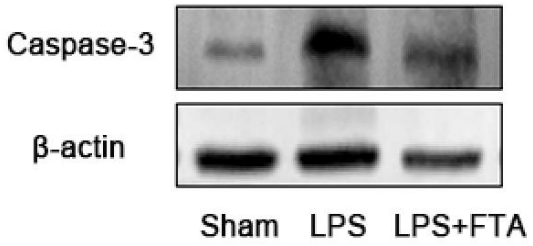

Loading order: Sham 1, LPS 1, FTA+LPS 1, Sham 2, LPS 2, FTA+LPS 2, Sham 3, LPS 3, FTA+LPS 3, marker.

Caspase-3

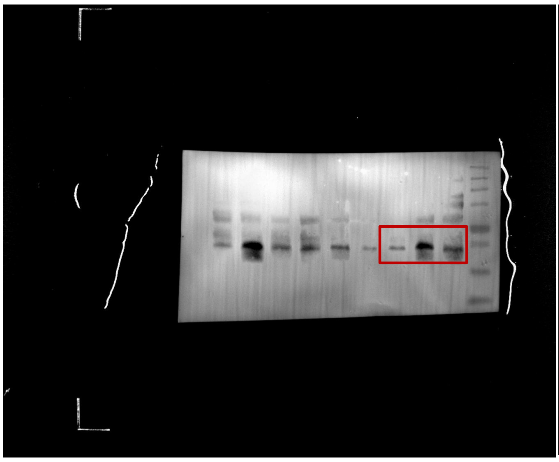

β-actin

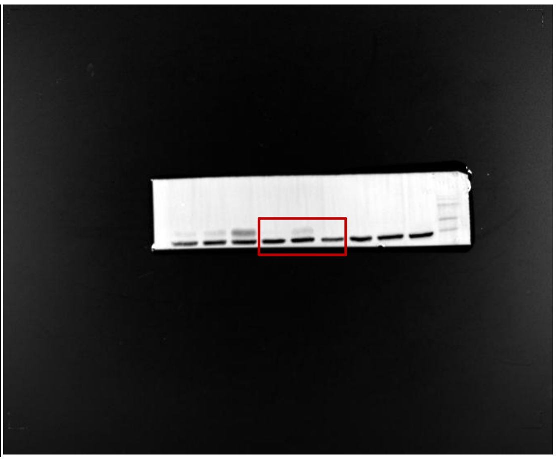

Figure 4A

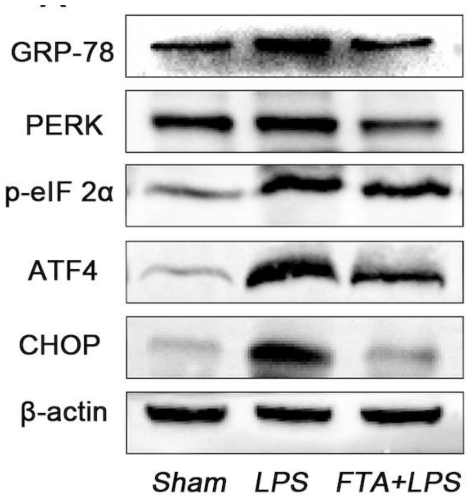

1. GRP78 （75kDa）

Loading order: Sham 1, LPS 1, FTA+LPS 1, marker, Sham 2, LPS 2, FTA+LPS 2, marker.

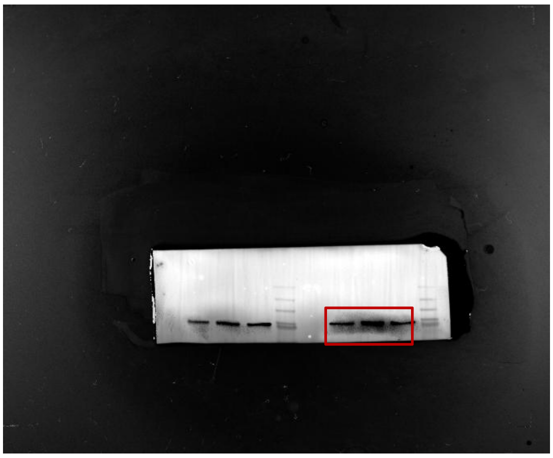
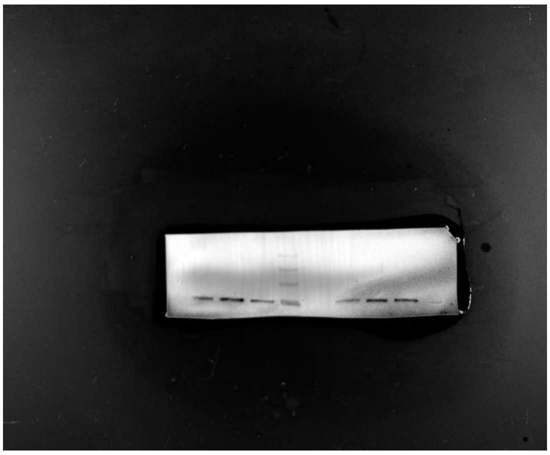

2. PERK（140kDa）

Loading order：Sham 1, LPS 1, FTA+LPS 1, marker, Sham 2, LPS 2, FTA+LPS 2, marker.

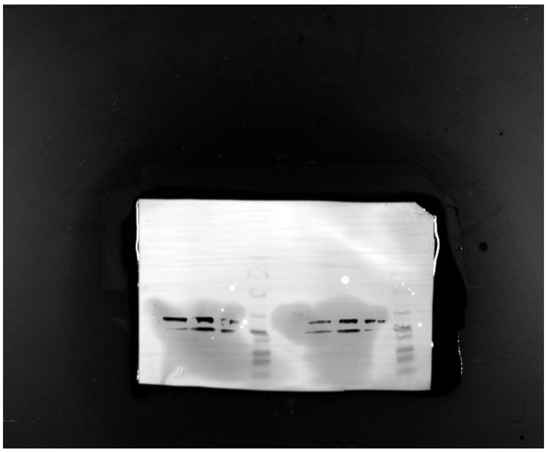
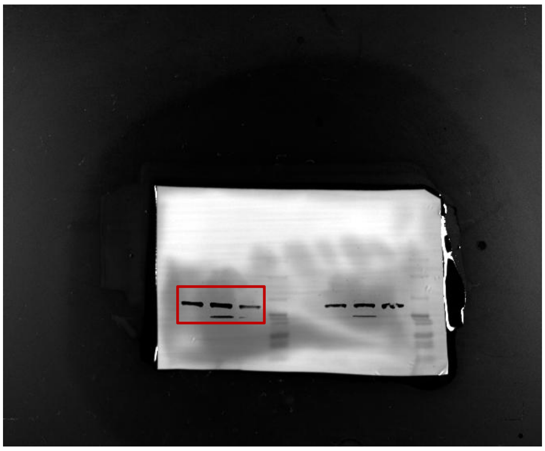

3. P-eIF 2α（38kDa）

Loading order：Sham 1, LPS 1, FTA+LPS 1, Sham 2, LPS 2, FTA+LPS 2, Sham 3, LPS 3, FTA+LPS 3, marker.

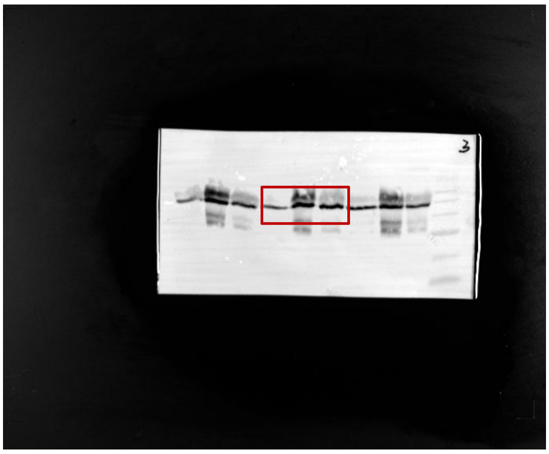

4. ATF4（39kDa）

Loading order：Sham 1, LPS 1, FTA+LPS 1, Sham 2, LPS 2, FTA+LPS 2, Sham 3, LPS 3, FTA+LPS 3, marker.

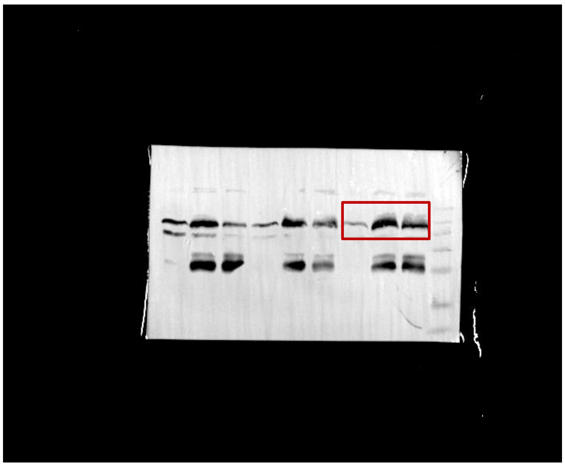

5. CHOP（27kDa）

Loading order：Sham 1, LPS 1, FTA+LPS 1, Sham 2, LPS 2, FTA+LPS 2, Sham 3, LPS 3, FTA+LPS 3, marker.

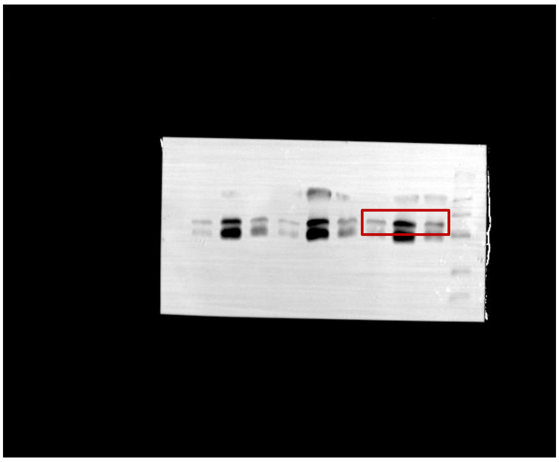

6. β-actin（43kDa）

Loading order：Sham 1, LPS 1, FTA+LPS 1, Sham 2, LPS 2, FTA+LPS 2, Sham 3, LPS 3, FTA+LPS 3, marker.

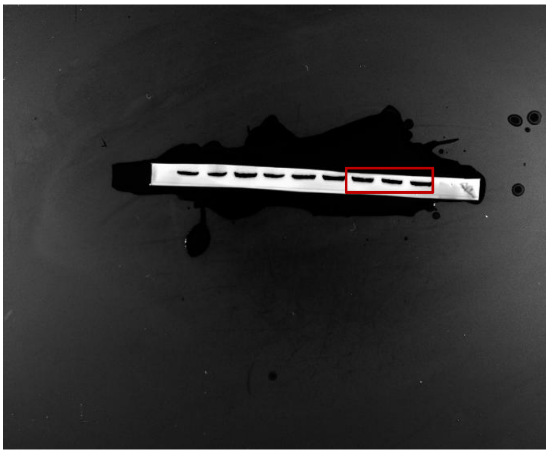

Supplement: Supplementary file 1 — Additional file 1. [file 12906_2023_3855_MOESM1_ESM.pdf]
